# Mixed-carbene cyclometalated iridium complexes with saturated blue luminescence†
†Electronic supplementary information (ESI) available: Experimental details, X-ray crystallography summary table, UV-vis excitation spectra, and NMR spectra. CCDC 1879106 and 1879107. For ESI and crystallographic data in CIF or other electronic format see DOI: 10.1039/c9sc01386e


**DOI:** 10.1039/c9sc01386e

**Published:** 2019-05-27

**Authors:** Hanah Na, Louise M. Cañada, Zhili Wen, Judy I-Chia Wu, Thomas S. Teets

**Affiliations:** a Department of Chemistry , University of Houston , 3585 Cullen Blvd. Room 112 , Houston , TX , USA 77204-5003 . Email: tteets@uh.edu

## Abstract

Cyclometalated iridium complexes have emerged as top-performing emitters in organic light-emitting diodes (OLEDs) and other optoelectronic devices. A persistent challenge has been the development of cyclometalated iridium complexes with deep blue luminescence that have the requisite color purity, efficiency, and stability to function in color displays. In this work we report a new class of cyclometalated iridium complexes with saturated blue luminescence. These complexes have the general structure Ir(C^C:^NHC^)_2_(C^C:^ADC^), where C^C:^NHC^ is an N-heterocyclic carbene (NHC) derived cyclometalating ligand and C^C:^ADC^ is a different type of cyclometalating ligand featuring an acyclic diaminocarbene (ADC). The complexes are prepared by a cascade reaction that involves nucleophilic addition of propylamine to an isocyanide precursor followed by base-assisted cyclometalation of the ADC intermediate. All three emit deep blue light with good quantum efficiencies (*Φ*_PL_ = 0.13–0.48) and color profiles very close to the ideal primary blue standards for color displays.

## Abstract 2

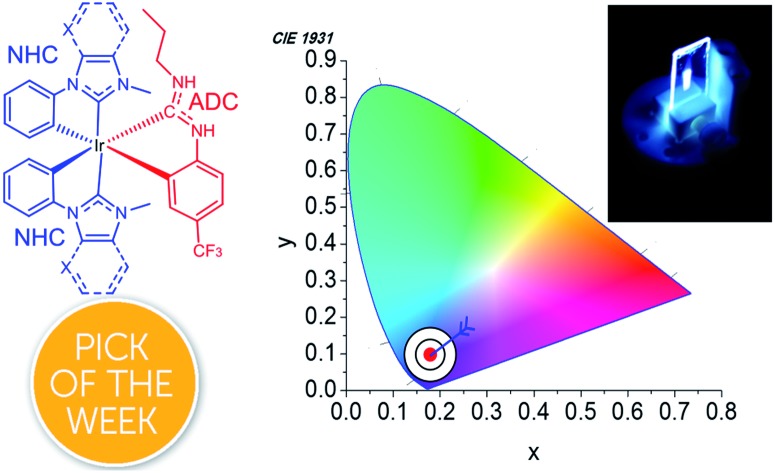
A new structural class of mixed-carbene cyclometalated iridium complexes with intense, high-purity blue luminescence are described.

## Introduction

**Fig. 1:**
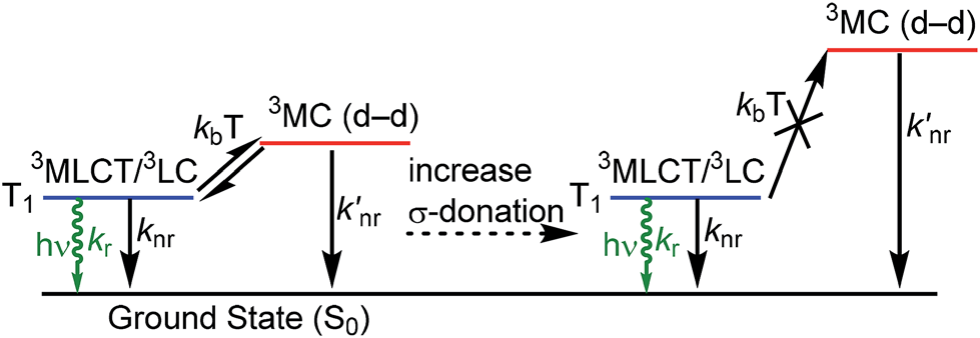
Partial excited-state diagram for cyclometalated iridium complexes, showing partial occupation of ^3^MC states and destabilization of these states when stronger σ-donor ligands are incorporated.

Organometallic compounds which luminesce from triplet excited states, *i.e. via* phosphorescence, have become prominent in a number of applications. Cyclometalated iridium complexes^1^ are a class of molecular phosphors that have achieved commercial success in organic light-emitting diodes (OLEDs)^1^–^5^ and have also been demonstrated to be effective in other contexts such as sensing^6^,^7^ and photocatalysis.^8^,^9^ Photoluminescence attributes of cyclometalated iridium complexes include high quantum yields, short lifetimes, and facile color tunability. A significant fundamental challenge in the design of luminescent iridium complexes, which is especially important in OLED research, is the lack of deep blue phosphorescent compounds with adequate performance metrics – *i.e.* color purity, quantum efficiency, and stability – to function in color displays or other optoelectronic applications. The physical origins of this challenge are summarized in Fig. 1. The HOMO in cyclometalated iridium complexes usually involves significant contribution from an Ir-centered dπ orbital, *i.e.* a t_2g_ orbital in rigorously *O*_h_ complexes, whereas the LUMO is an unoccupied π* orbital on the cyclometalating ligands. These complexes luminesce from excited states that have contributions from triplet ligand-centered (^3^LC) states localized on the cyclometalating ligand and triplet metal-to-ligand charge transfer (^3^MLCT) states, which mix through configuration interaction in the low-energy T_1_ state.^1^ The lowest-energy ligand-field or metal-centered state, which involves transitions between Ir-centered d orbitals, is also a triplet state (^3^MC).^10^ One of the reasons bis-cyclometalated iridium complexes are very successful as triplet emitters is that the ^3^MC state is typically much higher in energy than the T_1_ state and thus cannot be populated. However, in deep-blue-emitting compounds with high-energy T_1_ states, the ^3^MC state is thermally accessible, and population of this nonradiative state decreases quantum yield and also promotes electrons into metal–ligand σ* orbitals, inducing ligand-loss decomposition pathways that limit photostability and lead to degradation of devices fabricated with these emitters.^11^

As is also shown in Fig. 1, incorporation of strong σ-donor ligand sets can destabilize the deleterious ^3^MC state, allowing efficient luminescence from T_1_ and greater photostability. In an ideal case, where the σ-donor only perturbs the unoccupied dσ* orbitals and does not influence the frontier orbitals involved in the luminescent T_1_ state, this perturbation of the ^3^MC state would not influence the photoluminescence spectrum but should improve the quantum yield and photostability. As a result, N-heterocyclic carbenes (NHCs) have emerged as popular design elements in blue-emitting cyclometalated iridium complexes. Many recent works describe cyclometalated iridium complexes with NHC-containing C^C: cyclometalating ligands, chelated to iridium through the NHC and a phenyl group.^12^–^21^ In the context of blue OLEDs a majority of the recent breakthroughs involve homoleptic tris-cyclometalated Ir(C^C:^NHC^)_3_ complexes.^12^,^14^,^15^,^17^,^19^ The synthetic chemistry of heteroleptic compounds with only two C^C:^NHC^ cyclometalating ligands is much less developed, although there are a couple of examples of blue-emitting bis-cyclometalated complexes of the type Ir(C^C:^NHC^)_2_(L^X), where L^X is a pyrazolate or triazolate-based ancillary ligand.^16^,^18^

Until recently NHCs were the only type of carbene ligand incorporated into blue-emitting iridium complexes. We recognized the possibility that acyclic diaminocarbenes (ADCs), which are known to be even stronger σ-donors than NHCs on account of the greater 2p character in their σ orbital,^22^–^25^ could potentially destabilize ^3^MC states to an even greater extent than is possible with NHCs and improve the photostability of blue-phosphorescent complexes. We have shown that ADCs can be installed onto cyclometalated iridium complexes *via* on-complex, nucleophilic addition to coordinated isocyanides. Versions of these complexes exhibit intense blue luminescence when immobilized in polymer films,^26^–^28^ with quantum efficiencies as high as 79% for a cyclometalated ADC complex.^27^ However, the biggest limitation of these first-generation compounds, which will preclude any practical device applications, is their rather poor color purity, with significant sky blue to blue-green coloration in the photoluminescence. We observed CIE coordinates^29^ (CIE*x*, CIE*y*) = (0.20, 0.41) for complexes with cyclometalated ADC ligands,^27^ with (CIE*x*, CIE*y*) = (0.16, 0.21) and (0.17, 0.28) for blue-emitting compounds with “Chugaev-type” bis(ADC) ancillary ligands.^26^,^28^ The CIE*y* coordinates in particular are larger than the ideal values (<0.1) for high-purity blue emission; the most widely used industry standards are the International Electrotechnical Commission (IEC) sRGB standard, (CIE*x*, CIE*y*) = (0.15, 0.06), and the primary blue standards proscribed by National Television System Committee (NTSC) (CIE*x*, CIE*y*) = (0.14, 0.08) and Society of Motion Picture and Television Engineers (SMPTE-C) (CIE*x*, CIE*y*) = (0.155, 0.07). In addition, the blue-emitting ADC-containing complexes we prepared all use the C^N cyclometalating ligand 2-(2,4-difluorophenyl)pyridine (F_2_ppy), which is known to degrade in OLED devices *via* ligand defluorination.^11^ In the present work, we address these two limitations and improve upon our previous designs, introducing three new compounds that are devoid of sp^2^ C–F bonds and give rise to high-purity blue photoluminescence. These mixed-carbene compounds have the general formula Ir(C^C:^NHC^)_2_(C^C:^ADC^), where C^C:^NHC^ is an N-heterocyclic carbene (NHC) derived cyclometalating ligand and C^C:^ADC^ an ADC-containing cyclometalating ligand. These compounds expand the synthetic chemistry of heteroleptic NHC-derived cyclometalated iridium complexes and show that ADC ligands and NHC ligands can be partnered on the same complex. These compounds exhibit very promising photoluminescence attributes, in particular CIE coordinates that are very close to NTSC and IEC standards for color displays, making these compounds excellent candidates for incorporation into OLEDs.

## Results and discussion

### Synthesis and characterization

**Scheme 1:**
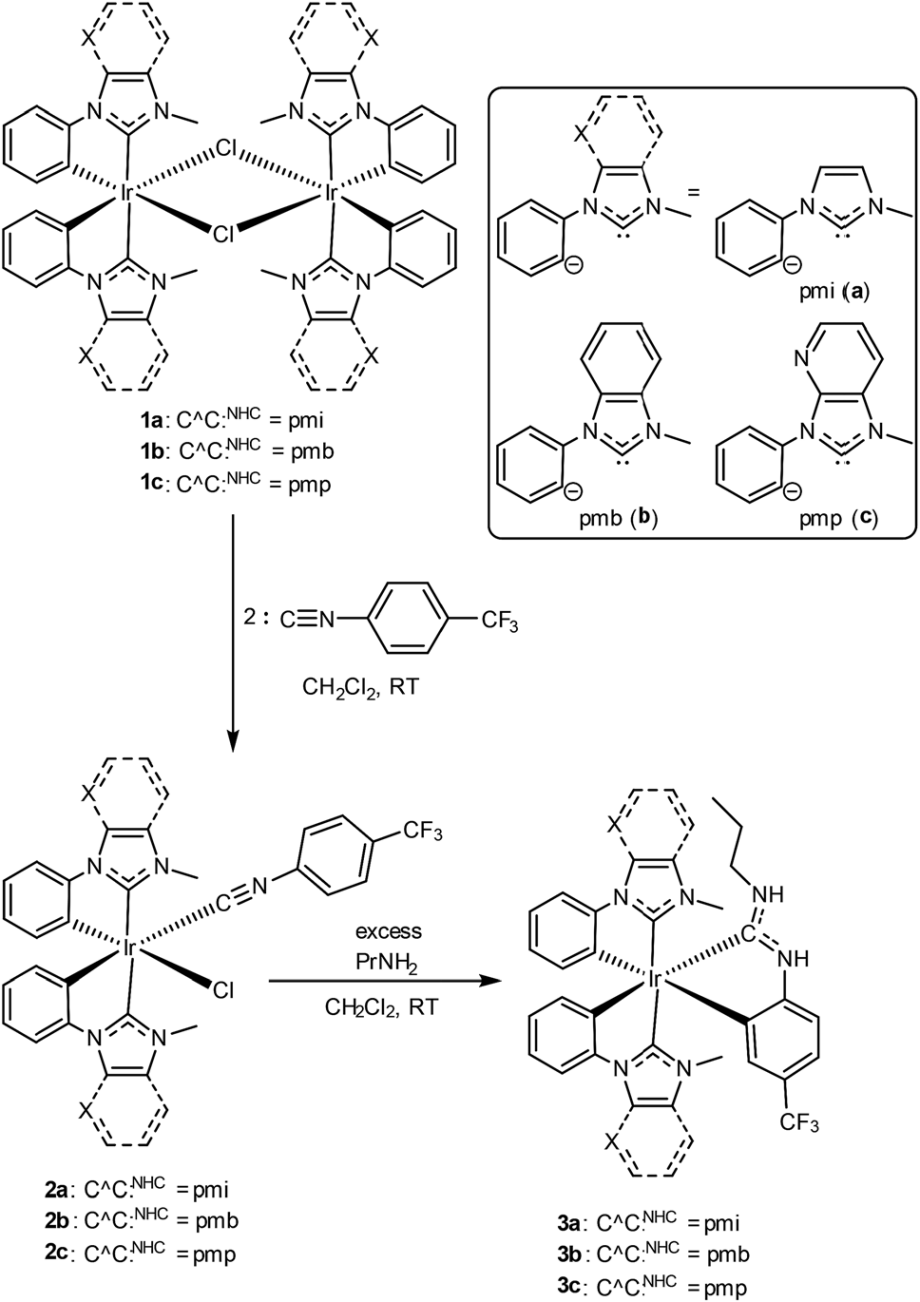
Synthetic procedure to prepare blue-emitting complexes **3a–c**.

Scheme 1 outlines the synthesis of the compounds described in this work. Preparation of the mixed-carbene complexes begins with precursors of the type [Ir(C^C:^NHC^)_2_(μ-Cl)]_2_ (**1a–c**); two of these (**1a** and **1b**) have been previously described.^16^ Treatment of dimers **1a–c** with 4-trifluoromethylphenyl isocyanide forms mononuclear complexes of the type Ir(C^C:^NHC^)_2_(CN-*p*-C_6_H_4_CF_3_)(Cl) (**2a–c**), which then are treated with excess propylamine to form the final ADC-containing complexes **3a–c**. Complexes **3a–c** form *via* a cascade reaction involving metal-mediated nucleophilic addition of the amine to the coordinated isocyanide, followed by base-assisted cyclometalation to form the final neutral, tris-chelated heteroleptic products. This unconventional synthetic approach, which we introduced recently in a set of C^N-cyclometalated complexes,^27^ allows us to generate ADC-containing products that are not accessible using traditional ligand substitution or transmetalation approaches. The identity and purity of the final products were ascertained by ^1^H, ^19^F, and ^13^C{^1^H} NMR spectroscopy. Chemical inequivalency of the NHC's CH_3_ resonances (^1^H and ^13^C{^1^H}) and two sets of aromatic ^1^H signals for the two C^C:^NHC^ ligands evince *C*_1_ symmetry, and characteristic resonances of the ADC, especially the propyl group and the two inequivalent N–H protons in the ^1^H spectrum and a downfield ^13^C{^1^H} resonance ∼200 ppm, are easily located in each case.

**Fig. 2:**
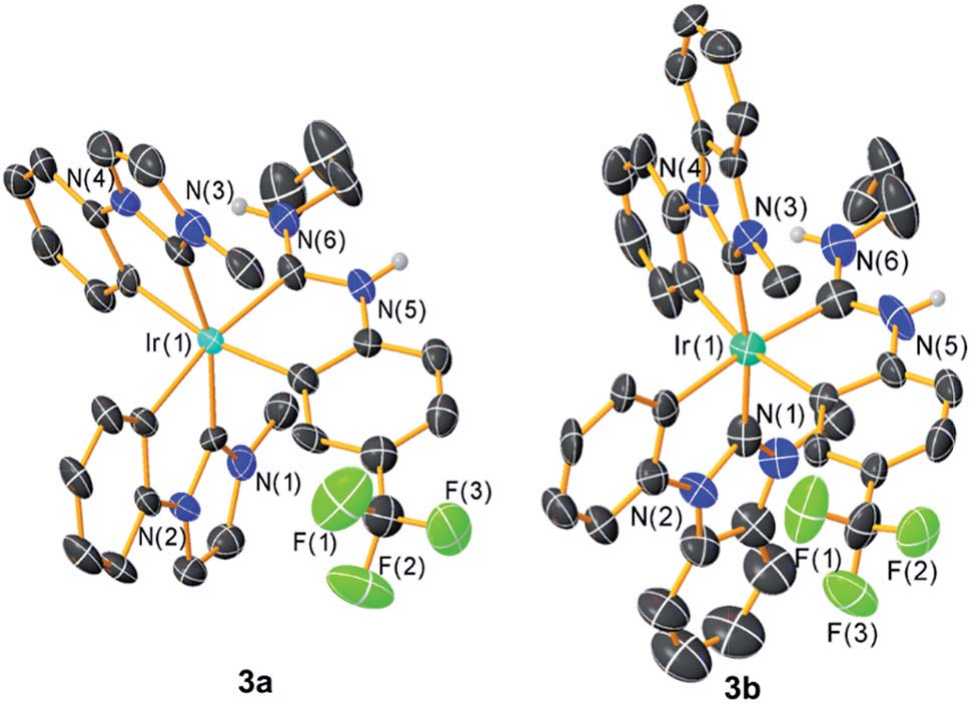
Crystal structures of complexes **3a** and **3b**, with ellipsoids shown at the 50% probability level. Carbon atoms are shown in black and unlabeled, and carbon-bound hydrogen atoms are omitted for clarity.

Further confirmation of the structures of **3a–c** comes from single-crystal X-ray diffraction studies. Whereas the structure of **3c** could be solved but refined poorly, structures of **3a**‡
‡**3a**: CCDC 1879106, C_31_H_30_F_3_IrN_6_, *M* = 735.81, triclinic, *P*1[combining macron], *a* = 14.228(5) Å, *b* = 15.407(6) Å, *c* = 16.166(6) Å; *α* = 86.526(4)°, *β* = 65.199(4)°, *γ* = 85.672(4)°, *Z* = 4, 36 765 tot. refln., 12 993 ind. refln., *R*_int_ = 0.058, *R*_1_ = 0.098, w*R*_2_ = 0.300. and **3b**§
§**3b**: CCDC 1879107, C_39_H_34_F_3_IrN_6_, *M* = 835.92, monoclinic, *C*2/*c*, *a* = 27.270(8) Å, *b* = 20.718(6) Å, *c* = 14.832(4) Å; *β* = 116.007(3)°, *Z* = 8, 31 630 tot. refln., 7309 ind. refln., *R*_int_ = 0.047, *R*_1_ = 0.088, w*R*_2_ = 0.211. refined reasonably well and are included here in Fig. 2. X-ray crystallography confirms the pseudo-octahedral coordination geometry and *trans* arrangement of the NHC ligands and also verifies the chelated nature of the ADC ancillary ligand. Bond metrics of the ADC ligand, in particular the N–C bond distances and the carbene N–C–N bond angles, are very similar to the bond metrics observed in previously described complexes from our group with the same ancillary ligand partnered with pyridine- or thiazole-based C^N cyclometalating ligands.^27^ There are also not any substantial differences between Ir–C(NHC) and Ir–C(ADC) distances in these structures, with the latter distances falling in the range spanned by the Ir–C(NHC) distances.

### Electrochemistry

**Fig. 3:**
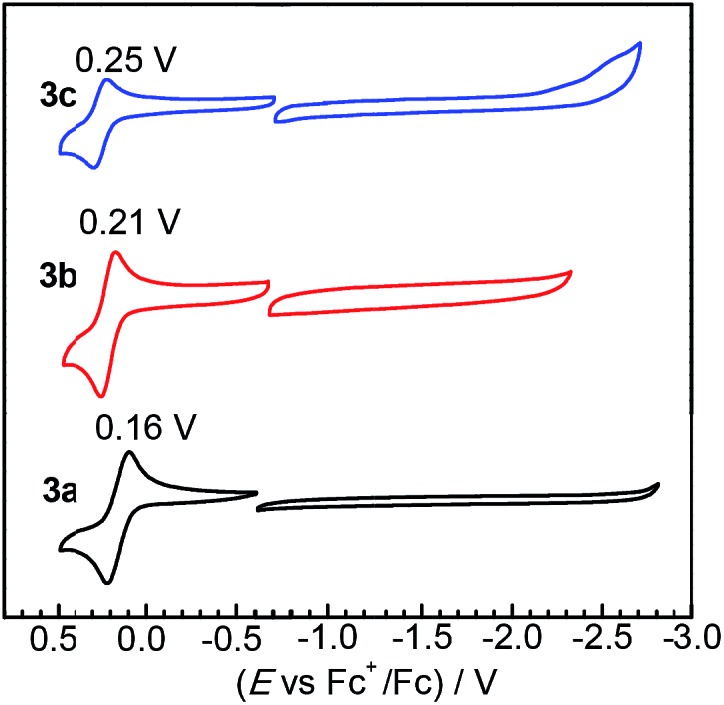
Overlaid cyclic voltammograms of **3a–c**, recorded in acetonitrile with 0.1 M NBu_4_PF_6_ electrolyte. A glassy carbon working electrode, platinum wire counter electrode, and silver wire pseudoreference electrode were used during the measurements, and potentials are referenced to an internal standard of ferrocene. Separate anodic (positive) and cathodic (negative) scans were recorded. Half-wave potentials (*E*_1/2_) are indicated above each oxidation wave, and currents are normalized to bring the plots onto the same scale.

The redox properties of complexes **3a–c** were investigated by cyclic voltammetry, which reveal that substituting NHC ligands with ADCs results in very small perturbations of frontier orbital energies. Fig. 3 overlays the cyclic voltammograms of **3a–c**, recorded in MeCN with 0.1 M NBu_4_PF_6_ electrolyte. All three complexes exhibit reversible one-electron Ir^IV^/Ir^III^ couples upon sweeping anodically. Sweeping in the negative direction, complexes **3a** and **3b** do not show any discernible reduction waves within the solvent window, whereas **3c** has an irreversible reduction at *ca.* –2.60 V *vs.* the Fc^+^/Fc couple. Compared to our previously reported complexes Ir(C^N)_2_(C^C:^ADC^) with nitrogen-containing cyclometalating ligands,^27^ complexes **3a–c** are easier to oxidize by at least 160 mV, signifying a destabilized HOMO in these complexes. To contextualize these results further we can compare the potentials to homoleptic *mer*-Ir(C^C:^NHC^)_3_ complexes, which likewise place the two NHCs *trans* to one another but have another cyclometalated NHC occupying the remaining coordination sites. Replacing one NHC cyclometalating ligand with the ADC in **3a–c** has a very small impact on the electrochemical potentials. Complex **3a** is oxidized at 0.16 V, which is almost identical to the complex *mer*-Ir(pmi)_3_ (*E*_1/2_ = 0.14 V).^13^ Similarly, the oxidation potentials for the pairs **3b** (*E*_1/2_ = 0.21 V) and *mer*-Ir(pmb)_3_ (*E*_1/2_ = 0.31 V)^13^ and **3c** (*E*_1/2_ = 0.25 V) and *mer*-Ir(pmp)_3_ (*E*_1/2_ = 0.23 V)^14^ are within 100 mV of one another. The homoleptic *mer*-Ir(C^C:^NHC^)_3_ complexes and new complexes **3a–c** are all very difficult to reduce, signifying very high-energy LUMOs that are responsible for the large HOMO–LUMO gaps in these C^C: cyclometalated compounds.

### Photophysical properties

**Fig. 4:**
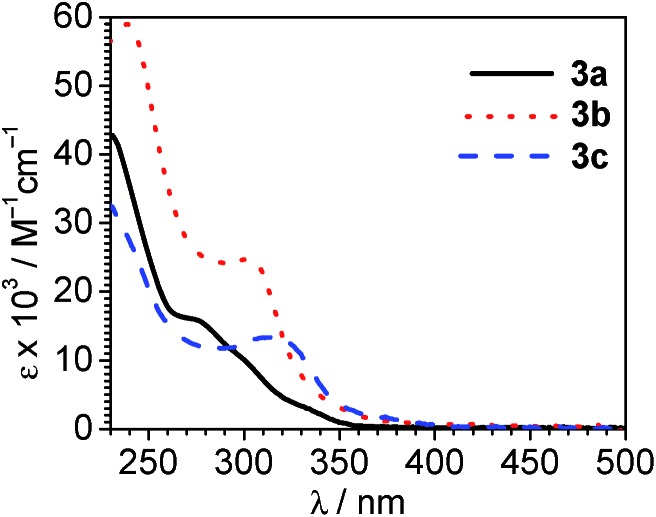
Overlaid UV-vis absorption spectra of **3a–3c**, recorded at room temperature in CH_2_Cl_2_.

UV-vis absorption spectra, recorded at room temperature in CH_2_Cl_2_, are collected in Fig. 4. Mixed-carbene complexes **3a–3c** are all colorless and only absorb in the UV region of the spectrum. All show intense absorption in the region of *λ* < 300 nm, consistent with ligand-centered π → π* transitions involving the NHC and/or ADC ligand. Consistent with the increased π conjugation of the benzo-fused pmb ligand, these UV absorption bands are more intense and less deep in the UV for complex **3b** (C^C:^NHC^ = pmb) compared to complex **3a** (C^C:^NHC^ = pmi). A weaker set of overlapping absorption bands is also observed, tailing to the low-energy reaches of the absorption profile. The overlapping bands include distinct maxima in **3b** (*λ* = 302 nm) and **3c** (*λ* = 315 nm), with multiple shoulders observed in each case. These absorption features are assigned to a combination of spin-allowed and spin-forbidden ^1^MLCT and ^3^MLCT transitions, as typically observed in cyclometalated iridium complexes. However, the destabilized LUMOs in these compounds, apparent from the cyclic voltammetry discussed above, result in these MLCT transitions occurring a higher energy than is typically observed in cyclometalated iridium complexes with C^N cyclometalating ligands.^3^,^30^ Cyclic voltammetry (Fig. 3) suggests that the LUMO in pyridyl-substituted complex **3c** is lower energy than those of **3a** and **3b**, and in line with that observation we see that the MLCT bands in **3c** occur at lower energy (longer wavelength) than the others.

**Fig. 5:**
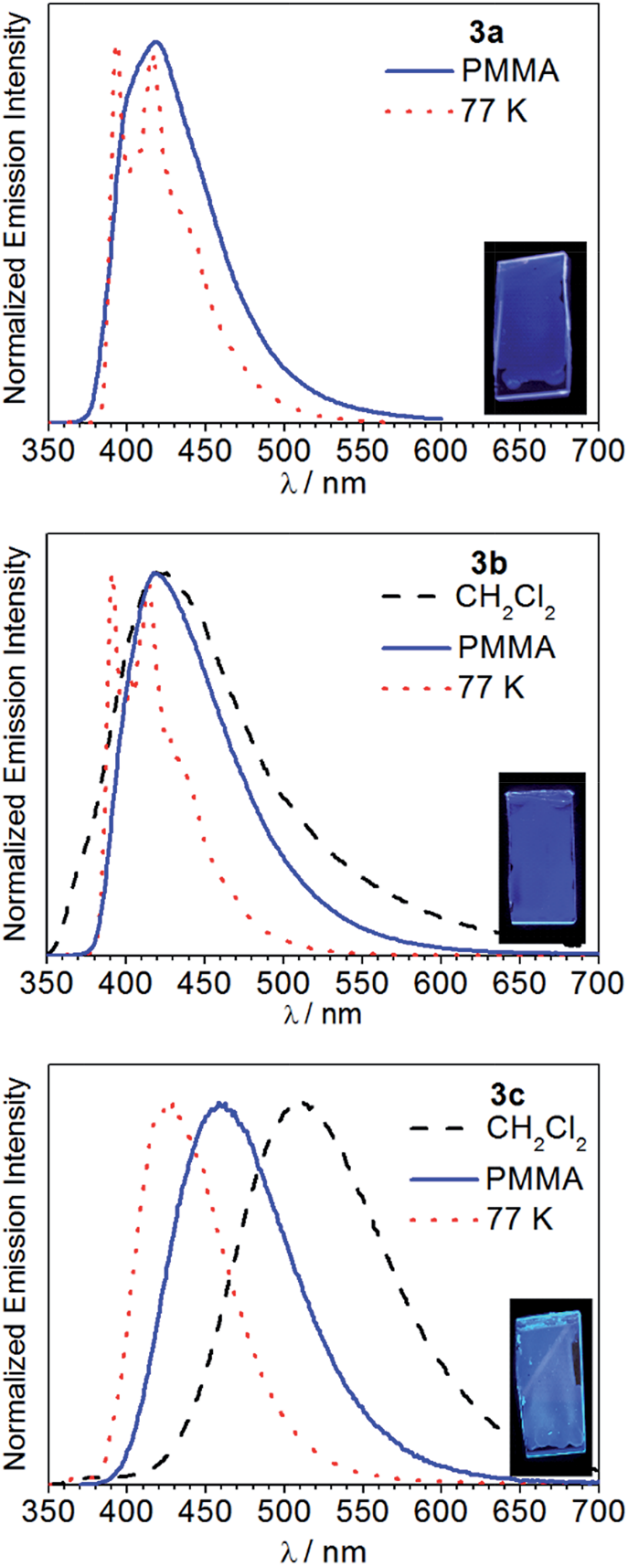
Photoluminescence spectra of **3a–c** recorded in CH_2_Cl_2_ at room temperature (black trace, dashed line), PMMA film at room temperature (blue trace, solid line), and in 1 : 3 CH_2_Cl_2_/toluene glass at 77 K (red trace, dotted line). Samples were excited at *λ* = 310 nm. The insets show photographs of the PMMA films under UV illumination.

Fig. 5 overlays the room-temperature photoluminescence spectra of complexes **3a–c**, whereas Table 1 summarizes the data. The complex Ir(pmi)_2_(C^C:^ADC^) (**3a**) is negligibly fluorescent in fluid solution, but when immobilized in poly(methyl methacrylate) (PMMA) at 2 wt% deep blue luminescence is observed, with a wavelength of maximum emission of 418 nm and a photoluminescence quantum yield (*Φ*_PL_) of 0.13. Replacing pmi with pmb in complex **3b** has no impact on the observed wavelength of emission, but in this case the quantum efficiency is much higher. In CH_2_Cl_2_ solution complex **3b** weakly emits, with a quantum yield of 0.013, and in PMMA the spectrum narrows and the quantum yield increases dramatically to 0.31. Finally, in complex **3c**, where the pyridyl-substituted cyclometalating ligand pmp is used, the photoluminescence bathochromically shifts and is environmentally dependent. In solution complex **3c** emits in the green region of the spectrum, with *λ* = 511 nm and a quantum yield of 0.39. The photoluminescence blue shifts significantly in PMMA film, with *λ* = 459 nm, and the quantum yield increases to 0.48. The photoluminescence lifetimes also vary across the series. When measured in PMMA film, the lifetimes decrease in the order **3a** (*τ* = 6.1 μs), **3b** (*τ* = 1.8 μs), and **3c** (*τ* = 0.85 μs). These differences in lifetimes and quantum yields are primarily driven by differences in *k*_r_, which increase in the order **3a** < **3b** < **3c**. Emission spectra were also recorded at 77 K in rigid solvent glass, and under these conditions the luminescence maximum displays a significant rigidochromic blue-shift in each case, signifying substantial charge-transfer. The spectra of **3a** and **3b** are much sharper with pronounced vibronic structure at 77 K, consistent with significant ligand-centered ^3^ππ* excited-state character as well, whereas the spectrum of **3c** is devoid of vibronic structure even at 77 K, indicating a T_1_ state that is mainly a metal-to-ligand charge transfer (^3^MLCT) state.

**Table 1:** Summary of photoluminescence data for **3a–c**^*a*^

	CH_2_Cl_2_, RT		77^*c*^ K	PMMA, RT^*d*^				
	*λ*/nm	*Φ*_PL_	*λ*/nm	*λ*/nm	*Φ*_PL_ ^*e*^	τ/μs	*k*_r_, *k*_nr_ × 10^–5^/s^–1^	(CIE*x*, CIE*y*)^*f*^
**3a**	N.A.^*b*^	N.A.^*b*^	393, 417	418	0.13(1)	6.1	0.21, 1.4	(0.16, 0.07)
**3b**	420	0.013	391, 415	418	0.31(1)	1.8	1.7, 3.8	(0.16, 0.10)
**3c**	511	0.39	430	459	0.48(7)	0.85	5.6, 6.1	(0.16, 0.18)

^*a*^For steady-state measurements, including quantum yields, *λ*_exc_ = 310 nm. For lifetime measurements, *λ*_exc_ = 330 nm.

^*b*^Not luminescent in solution at RT.

^*c*^Recorded in 1 : 3 CH_2_Cl_2_/toluene glass.

^*d*^2 wt% complex.

^*e*^Average of four or five independent trials, with standard deviation of the last digit shown in parentheses.

^*f*^From PMMA film data.

**Fig. 6:**
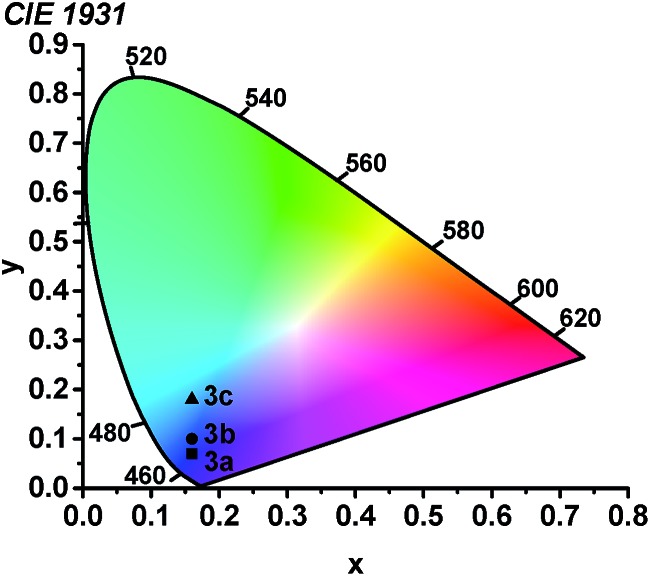
Chromaticity diagram showing the (CIE*x*, CIE*y*) coordinates of **3a–c**.

Using the thin-film photoluminescence data, CIE coordinates^29^ were determined and are summarized in Fig. 6. All three compounds fall in the blue region of the spectrum. The red-shifted emission in complex **3c** engendered by the pyridyl-substituted NHC results in significant sky-blue coloration with (CIE*x*, CIE*y*) coordinates of (0.16, 0.18). In contrast, the color profiles for **3a** and **3b** represent pure blue luminescence, with CIE coordinates of (0.16, 0.10) for **3b** and (0.16, 0.07) for **3a**. The color profiles of **3a–c** represent substantial improvements over our first two classes of cyclometalated iridium ADC complexes, all of which exhibited luminescence with significant sky blue to blue-green coloration rendering them unsuitable for device applications.^26^,^27^

### DFT calculations

**Fig. 7:**
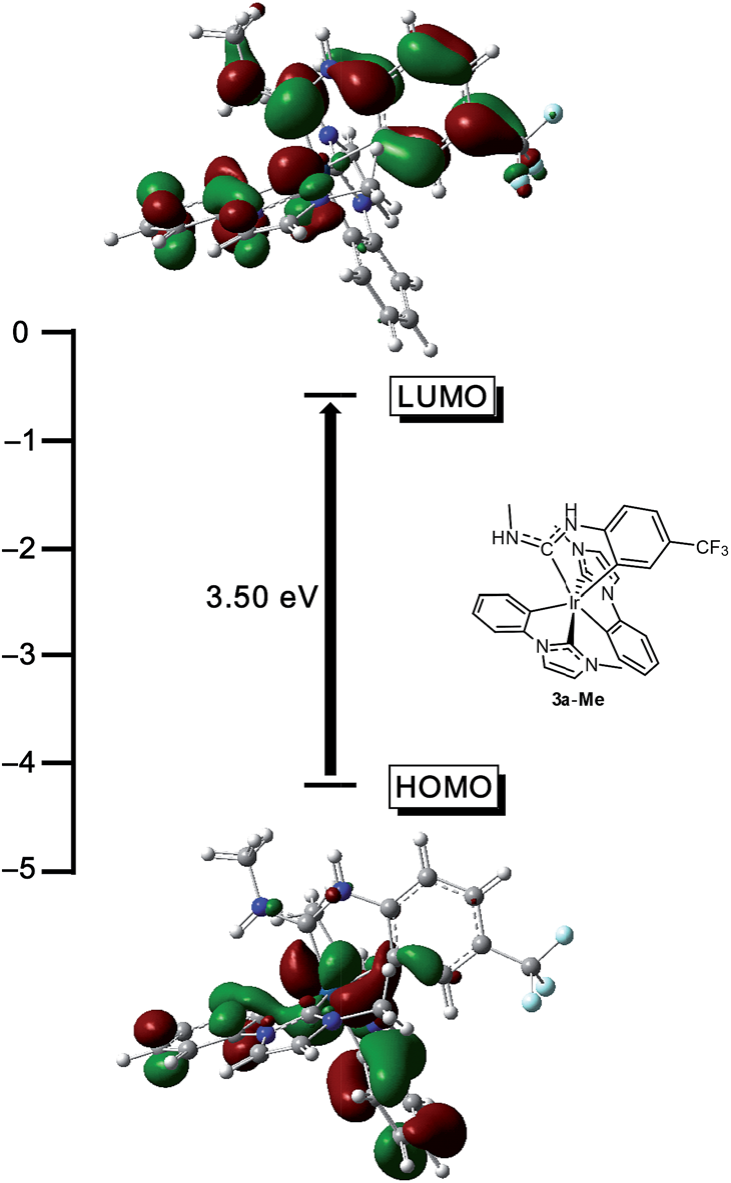
DFT-computed HOMO and LUMO energy values and plots for the model complex **3a-Me** (isovalue = 0.03 a.u.). Calculations were performed in the gas phase at MN15L/6-311G* for nonmetal atoms, with the SDD basis set used for Ir. The structure of **3a-Me** is also shown, in the approximate orientation used for the orbital depictions.

To validate the conclusions drawn from the experimental data presented above, and to determine the role of the ADC ligand in the frontier orbitals and triplet state, DFT calculations were performed on the model complex **3a-Me**, where the *N*-propyl group on the ADC is truncated to a methyl group. All calculations were performed in the gas phase at MN15L/6-311G* for nonmetal atoms, with the SDD basis set used for Ir. The optimized geometry is *C*_1_-symmetric with structural metrics very similar to the crystal structure (Fig. 2). In particular, the ADC N–C–N bond angle, which optimized to 116.75°, is intermediate between the angles of 116.1(15)° and 117.5(14)°, observed for the two crystallographically independent molecules of **3a**. Fig. 7 shows the computed HOMO and LUMO orbitals for **3a-Me**. The HOMO is a mixed metal–ligand orbital consisting primarily of contributions from Ir d orbitals and the phenyl rings of the cyclometalated NHC ligands, as is typical for carbene-based iridium cyclometalates.^13^,^15^ In contrast, the LUMO is almost exclusively ligand-centered, though it does involve contributions from one of the cyclometalated NHC ligands and the cyclometalated ADC ligand. A HOMO–LUMO gap of 3.50 eV was computed, consistent with the large electrochemical HOMO–LUMO gap (>3 eV) observed *via* cyclic voltammetry (Fig. 3).

**Fig. 8:**
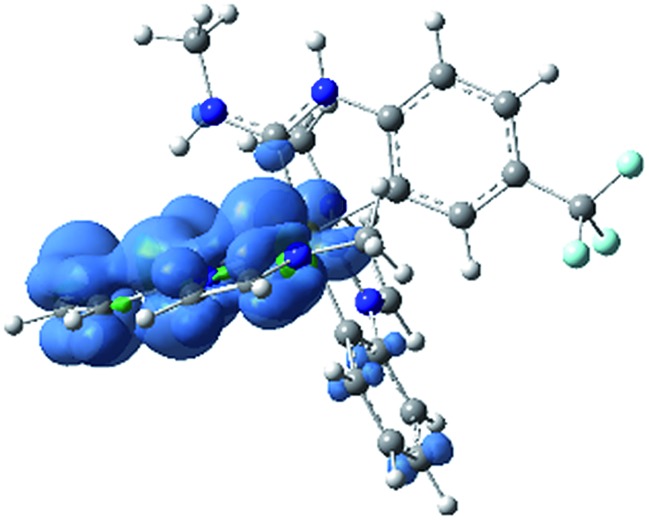
UDFT calculated triplet spin density of complex **3a-Me**. Contours are shown at an isovalue of 0.003 a.u. (blue indicates positive, green indicates negative).

In addition to ground-state calculations in the singlet spin state, the lowest-energy triplet state was computed using unrestricted DFT. There are no major geometric differences between the singlet ground state and the triplet excited state, and the computed singlet–triplet gap of 3.20 eV is a very good match for the *E*_0–0_ value of 3.15 eV, estimated from the first maximum in the 77 K photoluminescence spectrum of **3a** (Fig. 5). Fig. 8 shows the computed triplet spin density of **3a-Me**, with the unpaired spin density found to reside exclusively on the Ir center and one of the pmi ligands, consistent with the formulation of the T_1_ state as a mixed ^3^LC/^3^MLCT state. Notably, there is almost no spin density on the ADC ligand, even though the ADC is involved substantially in the LUMO. The nature of this triplet state is very similar to that of homoleptic Ir(C^C:^NHC^) complexes, consistent with the observation that the photoluminescence spectra of **3a–c** are quite similar to their homoleptic analogues (see below).

### Comparison of mixed-carbene and homoleptic NHC complexes

To further contextualize the photoluminescence properties and the CIE coordinates, we can compare complexes **3a–c** to homoleptic Ir(C^C:^NHC^)_3_ complexes which use the same three NHC ligands. Although Ir(C^C:^NHC^)_3_ complexes are available in both *fac* and *mer* isomers, with the stereochemistry not having a large influence on photoluminescence attributes,^13^,^14^ we focus here on comparisons to the *mer* isomer, since this isomer and **3a–c** include a *trans* arrangement of two of the NHC ligands. Immobilized in a thin film, compound **3c** exhibits nearly identical photoluminescence to that of *mer*-Ir(pmp)_3_,^14^ with the same featureless profile and nearly identical maximum wavelengths and CIE coordinates ((CIE*x*, CIE*y*) = (0.16, 0.16) for *mer*-Ir(pmp)_3_). The photoluminescence attributes of **3a** and **3b** are also quite similar to their respective *mer*-Ir(C^C:^NHC^)_3_ (C^C:^NHC^ = pmi, pmb) analogues. These compounds also emit with maxima near 400 nm, and although precise quantum yield values in polymer film were not reported, they were estimated to be greater than 0.1–0.2.^13^ The photoluminescence quantum yields of **3a** (*Φ* = 0.13) and **3b** (*Φ* = 0.31) are in the same range or greater, suggesting the photoluminescence efficiencies of **3a** and **3b** are very similar to or better than the homoleptic analogues. In addition, CIE coordinates for electroluminescent devices made from *mer*-Ir(pmb)_3_ were found to be (0.17, 0.06) and (0.17, 0.08),^12^ very similar to the color profile observed for the photoluminescence of **3a** and **3b**. One other point of comparison are homoleptic *mer*-Ir(C^C:^NHC^)_3_ complexes with CF_3_-substituted pmi ligands.^15^ The photoluminescence quantum yields of these compounds were measured in PMMA films, with one isomer having *Φ*_PL_ = 0.14 and another with *Φ*_PL_ = 0.47, similar values to those of **3a** and **3b**. CIE coordinates for devices made from these CF_3_-substituted Ir(pmi)_3_ analogues are also nearly identical to those observed for **3a** and **3b**. Taken together, these comparisons indicate that installing cyclometalated ADC ancillary ligands preserves the favorable photoluminescence characteristics of the carbene-based iridium cyclometalates that have been used in some of the top-performing blue devices.^12^,^14^,^15^ The subtle influence that the ADC has on the photoluminescence color when compared to homoleptic Ir(C^C:^NHC^)_3_ complexes suggests that the ADC-centered orbitals do not contribute substantially to the T_1_ state, which is born out in the DFT calculations described here.

## Conclusions

In this work we address the long-standing challenge of designing effective blue phosphors for OLED applications. The compounds we describe are heteroleptic tris-cyclometalated iridium complexes with mixed-carbene ligation, prepared from isocyanide precursors by a nucleophilic addition/cyclometalation cascade sequence. The photoluminescence attributes of these compounds are admirable. In PMMA thin films, deep blue luminescence is observed for two members of the series, with CIE coordinates that match well with industry standards for blue phosphors. The photoluminescence quantum yields are good and the lifetimes are in the microsecond range, also important criteria for device applications. In short, the pure blue photoluminescence of the mixed-carbene compounds describe here and the potential advantages offered by the ADC moiety motivate continued evaluation of **3a–c** and related analogues as dopants for blue OLEDs.

## Conflicts of interest

There are no conflicts to declare.

## Supplementary Material

Supplementary information

Crystal structure data

Infographic
