# Supplementary material for: Mixed-carbene cyclometalated iridium complexes with saturated blue luminescence
Source: Chem Sci. 2019 May 27;10(25):6254–60. doi: 10.1039/c9sc01386e (PMC6617658; doi:10.1039/c9sc01386e)
Supplement: Supplementary file 1 [file SC-010-C9SC01386E-s001.pdf]

Electronic Supplementary Material (ESI) for Chemical Science.
This journal is © The Royal Society of Chemistry 2019

Supplementary Information for

Mixed-Carbene Cyclometalated Iridium Complexes

with Saturated Blue Luminescence

Hanah Na, Louise M. Cañada, Zhili Wen, Judy I-Chia Wu, and Thomas S. Teets*

Department of Chemistry, University of Houston,

3585 Cullen Blvd. Room 112, Houston, TX 77204-5003, USA

email: tteets@uh.edu

Contents

Index	*Page*
Experimental Section	S2–S5
X-ray crystallographic summary table	S6
Excitation spectra of **3b** and **3c**	S7
NMR spectra for **3a, 3b**, and **3c**	S18–S12
DFT-optimized Cartesian coordinates	S13–S16
References	S16

Experimental Section

Materials. Reactions were performed in ambient conditions unless otherwise stated. Dry solvents were obtained from a Grubbs Solvent Purification System and degassed with argon. Starting materials and reagents, unless otherwise specified, were obtained from commercial sources and used without further purification. The chloro bridged Ir dimer $[Ir(C\^{}C:)_2(\mu\text{-}Cl)]_2$ (C^C: = pmi, pmb) were prepared by refluxing pmi or pmb with $IrCl_3{\cdot}xH_2O$ in 2-ethoxyethanol.[1] 1-phenyl-3-methylpyridoimidazolium iodide was prepared following a literature procedure.[2] 4-trifluoromethylphenyl isocyanide was prepared by formylation of 4-trifluoromethylaniline, followed by dehydration with $POCl_3$.[3]

Physical Methods. ^1H, $^{13}C\{^1H\}$, and ^{19}F NMR spectra were recorded at room temperature using a JEOL ECA-500 or ECA-600 NMR spectrometer. UV-vis absorption spectra were recorded in dichloromethane in screw-capped 1 cm quartz cuvettes using an Agilent Carey 8454 UV-vis spectrophotometer. Steady-state emission and excitation spectra were recorded using a Horiba FluoroMax-4 spectrofluorometer. To exclude air, samples for solution emission spectra were prepared in a nitrogen-filled glove-box using dry, deoxygenated solvents, and thin-film PMMA samples were kept under nitrogen until immediately before measurement. Samples for solution emission were housed in 1 cm quartz cuvettes with septum-sealed screw caps, and samples for low-temperature emission were contained in a custom quartz EPR tube with high-vacuum valve and immersed in liquid nitrogen using a finger Dewar. Emission quantum yields in solution were determined relative to a standard of quinine sulfate in 0.05 M sulfuric acid, which has a reported fluorescence quantum yield (Φ_F) of 0.52.[4] The quantum yields of complexes doped into poly(methyl methacrylate) (PMMA) thin films were recorded using a Spectralon-coated integrating sphere. Phosphorescence lifetimes were measured on a Horiba DeltaFlex Lifetime System, using 330 nm excitation. Cyclic voltammetry (CV) experiments were performed with a CH Instruments 602E potentiostat using a three-electrode system in a nitrogen-filled glove-box. A 3 mm diameter glassy-carbon electrode, Pt wire, and silver wire were used as working electrode, counter electrode, and pseudoreference electrode, respectively. Measurements were carried out in acetonitrile solution with 0.1 M $TBAPF_6$ as a supporting electrolyte at scan rate of 0.1 V/s. Ferrocene was used as an internal standard, and potentials were referenced to the ferrocene/ferrocenium couple.

PMMA film fabrication. A solution of PMMA (98 mg, 35 kDa) in dichloromethane (1.0 mL) was stirred at room temperature in a nitrogen-filled glove-box. Then, the respective iridium complex (2 mg, 2 wt. %) was added to the solution and stirred. The resulting solution was drop coated on a quartz substrate and dried at room temperature overnight.

Synthesis

Synthesis of $[Ir(pmp)_2(\mu\text{-}Cl)]_2$ (1c). Silver(I) oxide (113 mg, 0.49 mmol), 1-phenyl-3-methylpyridoimidazolium iodide (132 mg, 0.39 mmol) and $IrCl_3{\cdot}xH_2O$ (58 mg, 0.19 mmol) were dissolved in 5 mL of 2-ethoxyethanol under N_2. The reaction mixture was stirred and heated at 120 ° C for 24 h under N_2 while protected from light. The reaction mixture was cooled

to room temperature and concentrated under reduced pressure. Flash column chromatography on Celite using CH_2Cl_2 as the eluent was performed to remove the silver(I) salts. A brown oil was obtained, and addition of hexane gave a brown solid. It was further purified by flash column chromatography on silica gel using CH_2Cl_2/MeOH as the eluent. The tris-Ir(pmp)$_3$ was eluted with CH_2Cl_2 and then remaining crude product containing Ir dimer was eluted with CH_2Cl_2/MeOH. The dark-brown crude product was used without further purification. Yield: 9.2% (45 mg, 0.035 mmol).

Synthesis of Ir(pmi)$_2$(CNAr^{CF3})(Cl) (2a). [Ir(pmi)$_2$(μ-Cl)]$_2$ (145 mg, 0.134 mmol) was dissolved in 30 mL of CH_2Cl_2 inside the glove box. 4-trifluoromethylphenyl isocyanide (46 mg, 0.27 mmol) was added to the mixture. After 12 h of stirring at room temperature the solvent was removed under vacuum. The pale brown solid was washed with excess of hexane and dried under vacuum. Yield: 67% (125 mg, 0.18 mmol). ^{1}H NMR (600 MHz, $CDCl_3$): δ = 7.55 (t, *J* = 7.6 Hz, 3H), 7.43 (s, 1H), 7.29 (d, *J* = 8.2 Hz, 2H), 7.13 (s, 1H), 7.05 (d, *J* = 7.6 Hz, 2H), 7.01 (s, 1H), 6.83 (t, *J* = 7.6 Hz, 2H), 6.65 (d, *J* = 7.6 Hz, 1H), 6.56 (s, 1H), 6.44 (d, *J* = 7.6 Hz, 1H), 6.36 (d, *J* = 7.6 Hz, 1H), 4.38 (s, 3H), 4.09 (s, 3H).^{19}F NMR (470 MHz, $CDCl_3$): δ = –62.67 (s, CF_3, 3F).

Synthesis of Ir(pmb)$_2$(CNAr^{CF3})(Cl) (2b). [Ir(pmb)$_2$(μ-Cl)]$_2$ (100 mg, 0.078 mmol) was dissolved in 30 mL of CH_2Cl_2 inside the glove box. 4-trifluoromethylphenyl isocyanide was added (18 mg, 0.11 mmol) to the mixture. After 12 h of stirring at room temperature the solvent was removed under vacuum. The pale yellow solid was washed with excess of hexane and dried under vacuum. Yield: 77% (98 mg, 0.12 mmol). ^{1}H NMR (500 MHz, $CDCl_3$): δ = 8.18–8.14 (m, 2H), 7.74 (d, *J* = 7.4 Hz, 2H), 7.66 (d, *J* = 5.7 Hz, 1H), 7.58–7.55 (m, 3H), 7.49 (d, *J* = 4.0 Hz, 2H), 7.43 (d, *J* = 4.0 Hz, 2H), 7.32 (d, *J* = 8.0 Hz, 2H), 6.95–6.83 (m, 2H), 6.61 (t, *J* = 7.2 Hz, 1H), 6.51 (s, br, 2H), 6.43 (d, *J* = 7.5 Hz, 1H), 4.68 (s, 3H), 4.35 (s, 3H). ^{19}F NMR (470 MHz, $CDCl_3$): δ = –62.73 (s, CF_3, 3F).

Synthesis of Ir(pmp)$_2$(CNAr^{CF3})(Cl) (2c). [Ir(pmp)$_2$(μ-Cl)]$_2$ (45 mg, 0.035 mmol, crude product from previous step) was dissolved in 10 mL of CH_2Cl_2 inside the glove box. 4-trifluoromethylphenyl isocyanide was added (10 mg, 0.06 mmol) to the mixture. After 12 h of stirring at room temperature the solvent was removed under vacuum. The brown solid was washed with excess of hexane and dried under vacuum. The crude product was used without further purification or characterization. Yield: 61% (35 mg, 0.043 mmol).

General procedure for preparation of Ir(C^C:^{NHC})$_2$(C^C:^{ADC}) complexes 3a–3c. The complex Ir(C^C)$_2$(CNAr^{CF3})(Cl) (**2a–2c**) was dissolved in a minimum amount of CH_2Cl_2 and combined with excess propylamine. The resulting reaction mixture was stirred at room temperature for 4 d. The resulting solution was washed with water and organic layer was collected. The collected CH_2Cl_2 layer was passed through $MgSO_4$. The crude product was purified by flash short column chromatography on silica using CH_2Cl_2 as the eluent and further purified by recrystallization from CH_2Cl_2/hexane.

Ir(pmi)$_2$(CNH(Ar^{CF3})NHPr) (3a). Prepared by the general procedure using 100 mg (0.14

mmol) of Ir(pmi)$_2$(CNAr^{CF3})(Cl) (**2a**) and propylamine (3 mL) with a reaction time of 4 d. The crude product was purified by flash chromatography on a short column of silica using CH_2Cl_2 as the eluent and further purified by recrystallization from CH_2Cl_2/hexane to give an off-white solid. Yield: 49% (51 mg, 0.069 mmol). ^{1}H NMR (600 MHz, Acetone-d$_6$): δ = 9.77 (s, 1H), 7.72 (d, *J* = 2.1 Hz, 1H), 7.62 (d, *J* = 2.1 Hz, 1H), 7.21 (d, *J* = 7.6 Hz, 1H), 7.15 (d, *J* = 7.6 Hz, 1H), 7.07 (d, *J* = 1.4 Hz, 1H), 7.03 (d, *J* = 1.4 Hz, 1H), 7.01 (d, *J* = 2.1 Hz, 1H), 6.95–6.84 (m, 3H), 6.76 (td, *J* = 1.4, 7.6 Hz, 1H), 6.71–6.69 (m , 2H), 6.57 (t, *J* = 7.2 Hz, 1H), 6.52 (t, *J* = 6.9 Hz, 1H), 5.77 (s, 1H), 3.60 (s, 3H), 3.23–3.11 (m, 2H), 3.09 (s, 3H), 1.43–1.23 (m, 2H), 0.65 (t, *J* = 7.6 Hz, 3H). ^{13}C{^{1}H} NMR (151 MHz, Acetone-d$_6$): δ = 199.3, 174.8, 173.0, 158.5, 151.9, 151.3, 148.1, 147.1, 146.1, 138.8, 138.1, 134.5, 124.8, 124.3, 121.6, 120.8, 120.3, 119.2, 117.4, 117.3, 114.6, 114.2, 110.4, 110.1, 108.0, 43.8, 36.5, 35.9, 21.8, 10.3. ^{19}F NMR (470 MHz, Acetone-d$_6$): δ = −60.57 (s, 3F, CF_3).

Ir(pmb)$_2$(CNH(Ar^{CF3})NHPr) (3b). Prepared by the general procedure using 80 mg (0.098 mmol) of Ir(pmb)$_2$(CNAr^{CF3})(Cl) (**2b**) and propylamine (3 mL) with a reaction time of 4 d. The crude product was purified by flash chromatography on a short column of silica using CH_2Cl_2 as the eluent and further purified by recrystallization from CH_2Cl_2/hexane to give an off-white solid. Yield: 86% (70 mg, 0.084 mmol). ^{1}H NMR (500 MHz, Acetone-d$_6$): δ = 9.97 (s, 1H), 8.25 (dd, *J* = 23.5, 7.4 Hz, 2H), 7.86 (dd, *J* = 24.1, 7.4 Hz, 2H), 7.54 (dd, *J* = 18.6, 7.7 Hz, 2H), 7.39–7.27 (m, 4H), 7.01–6.98 (m, 2H), 6.94–6.90 (m, 3H), 6.86 (t, *J* = 7.4 Hz, 1H), 6.71 (d, *J* = 7.4 Hz, 1H), 6.58 (t, *J* = 7.7 Hz, 1H), 6.51 (t, *J* = 7.2 Hz, 1H), 5.85 (s, 1H), 3.86 (s, 3H), 3.40 (s, 3H), 3.28–3.23 (m, 1H), 3.16–3.12 (m, 1H), 1.36–1.24 (m, 2H), 0.61 (t, *J* = 7.4 Hz, 3H). ^{13}C{^{1}H} NMR (151 MHz, dmso-d$_6$): δ = 197.8, 188.2, 186.3, 159.0, 151.5, 151.0, 149.0, 148.2, 145.4, 138.4, 137.5, 136.7, 136.5, 133.7, 132.2, 132.1, 128.7, 126.9, 125.3, 125.1, 124.8, 123.7, 123.5, 122.7, 122.5, 121.4, 120.4, 118.5, 112.9, 112.5, 111.5, 111.4, 111.2, 109.5, 44.5, 33.8, 33.3, 21.9, 10.9. ^{19}F NMR (470 MHz, Acetone-d$_6$): δ = −60.79 (s, 3F, CF_3).

Ir(pmp)$_2$(CNH(Ar^{CF3})NHPr) (3c). Prepared by the general procedure using 30 mg (0.035 mmol) of Ir(pmb)$_2$(CNAr^{CF3})(Cl) (**2c**) and propylamine (1.2 mL) with a reaction time of 4 d. The crude product was purified by flash chromatography on a short column of silica using CH_2Cl_2 as the eluent and further purified by recrystallization from CH_2Cl_2/hexane to give an off-white solid. Yield: 49% (14 mg, 0.017 mmol). ^{1}H NMR (500 MHz, Acetone-d$_6$): δ = 10.00 (s, 1H), 8.78 (q, *J* = 7.8 Hz, 2H), 8.43 (d, *J* = 5.2 Hz, 1H), 8.35 (d, *J* = 5.2 Hz, 1H), 7.96 (d, *J* = 7.4 Hz, 1H), 7.91 (d, *J* = 8.0 Hz, 1H), 7.37 (t, *J* = 7.5 Hz, 1H), 7.29 (t, *J* = 7.5 Hz, 1H), 7.06 (s, 1H), 7.03–6.94 (m, 2H), 6.93–6.90 (m, 2H), 6.86 (t, *J* = 7.7 Hz, 1H), 6.72 (d, *J* = 7.5 Hz, 1H), 6.61 (t, *J* = 7.5 Hz, 1H), 6.55 (t, *J* = 7.5 Hz, 1H), 5.98 (s, 1H), 3.90 (s, 3H), 3.41 (s, 3H), 3.29–3.25 (m, 1H), 3.19–3.16 (m, 1H), 1.38–1.30 (m, 2H), 0.62 (t, *J* = 7.4 Hz, 3H). ^{13}C{^{1}H} NMR (151 MHz, dmso-d$_6$): δ = 196.8, 189.9, 187.9, 159.1, 150.5, 150.0, 148.0, 147.1, 146.1, 145.9, 144.2, 143.6, 143.2, 138.1, 137.2, 129.4, 129.3, 125.9, 125.3, 121.3, 120.3, 118.83, 118.79, 118.7, 118.3, 118.1, 114.8, 114.4, 109.7, 44.6, 34.1, 33.5, 21.9, 10.9. ^{19}F NMR (470 MHz, Acetone-d$_6$): δ = −60.86 (s, 3F, CF_3).

X-ray Crystallography Details. Single crystals were grown by vapor diffusion of hexane into concentrated CH_2Cl_2 solutions. Crystals were mounted on a Bruker Apex II three-circle

diffractometer using MoKα radiation (λ = 0.71073 Å). The data was collected at 123(2) K and was processed and refined within the APEXII software. Structures were solved by intrinsic phasing in SHELXT and refined by standard difference Fourier techniques in the program SHELXL.[5] Hydrogen atoms were placed in calculated positions using the standard riding model and refined isotropically; all non-hydrogen atoms were refined anisotropically. The crystals of both **3a** and **3b** were prone to desolvation upon removing from the mother liquor. As a result, both structures had large solvent-accessible voids that were excluded from the refinement via the SQUEEZE function in PLATON.[6] In addition, the desolvation caused some fracturing of the crystals, leading to some non-merohedral twinning that couldn't be resolved. As a result, Level A and B checkCIF alerts are noted in both structures, attributed to positive residual electron density near the Ir, and low C–C bond precision. CCDC 1879106 (**3a**), 1879107 (**3b**) contain the supplementary crystallographic data for this paper. These data are provided free of charge by the Cambridge Crystallographic Data Centre. Crystallographic details are summarized in Table S1.

Computational Details. Geometry optimizations for the singlet (ground state) and triplet (T_1 excited state) states of **3a-Me** were performed in the gas phase at the MN15L level employing Gaussian16. The 6-311G* basis set was used for C, H, N, and F, the SDD basis set with effective core potential (ECP) was used for Ir. Optimized geometries at both states matched well with the crystal structure. Vibrational frequency calculations verified the nature of the stationary points, indicating that both the singlet and triplet structures were minima on the potential energy surface. Spin density plots were computed by Multiwfn based on the **3a-Me** triplet state wavefunction.[7] Optimized Cartesian coordinates are listed in Tables S2 and S3.

Table S1. Crystallographic summary for **3a** and **3b**.

	**3a**	**3b**
CCDC	1879106	1879107
Crystal data		
Chemical formula	$C_{31}H_{30}F_3IrN_6$	$C_{39}H_{34}F_3IrN_6$
M_r	735.81	835.92
Crystal system, space group	Triclinic, $P\overline{1}$	Monoclinic, $C2/c$
a, b, c (Å)	14.228 (5), 15.407 (6), 16.166 (6)	27.270 (8), 20.718 (6), 14.832 (4)
α, β, γ (°)	86.526 (4), 65.199 (4), 85.672 (4)	90, 116.007 (3), 90
V (Å^3)	3206 (2)	7531 (4)
Z	4	8
μ (mm^{-1})	4.21	3.60
Crystal size (mm)	0.47 × 0.18 × 0.09	0.31 × 0.18 × 0.04
Data collection		
T_{min}, T_{max}	0.453, 0.746	0.428, 0.745
No. of measured, independent and observed [$I > 2\sigma(I)$] reflections	36765, 12993, 8435	31630, 7309, 5280
R_{int}	0.058	0.047
(sin θ/λ)$_{max}$ (Å^{-1})	0.625	0.617
Refinement		
$R[F^2 > 2\sigma(F^2)]$, $wR(F^2)$, S	0.098, 0.300, 1.05	0.088, 0.211, 1.08
No. of reflections	12993	7309
No. of parameters	745	449
No. of restraints	764	31
	$w = 1/[\sigma^2(F_o^2) + (0.1544P)^2 + 125.7637P]$ where $P = (F_o^2 + 2F_c^2)/3$	$w = 1/[\sigma^2(F_o^2) + 580.3679P]$ where $P = (F_o^2 + 2F_c^2)/3$
$\Delta\rho_{max}$, $\Delta\rho_{min}$ (e Å^{-3})	12.00, -4.95	6.47, -4.22

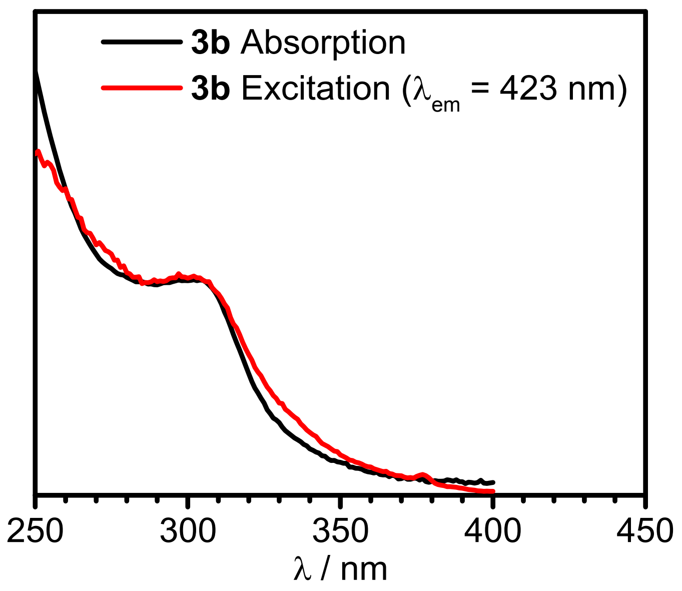

Figure S1. Overlaid UV-vis absorption and excitation spectra of **3b**, recorded in CH_2Cl_2 at room temperature.

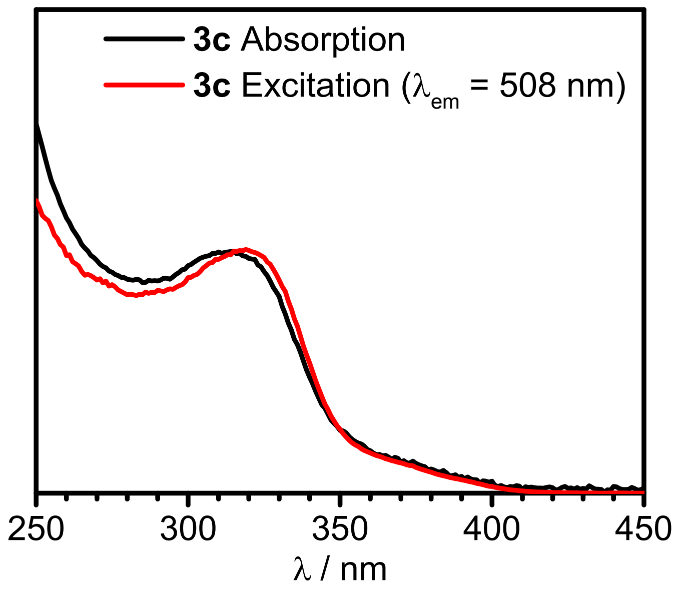

Figure S2. Overlaid UV-vis absorption and excitation spectra of **3c**, recorded in CH_2Cl_2 at room temperature.

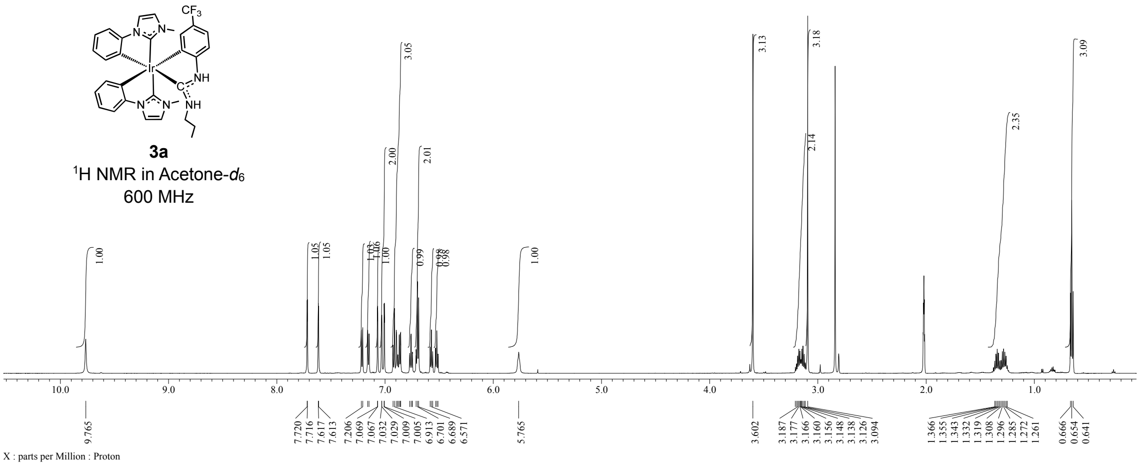

Figure S3. ^{1}H NMR spectrum of **3a**, recorded in acetone-*d*$_6$ at 600 MHz.

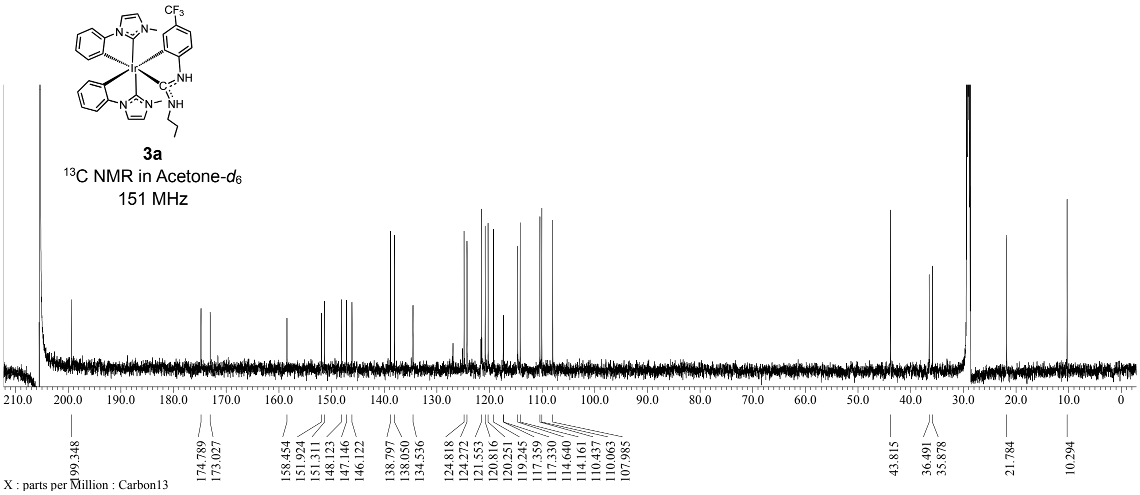

Figure S4. ^{13}C NMR spectrum of **3a**, recorded in acetone-*d*$_6$ at 151 MHz.

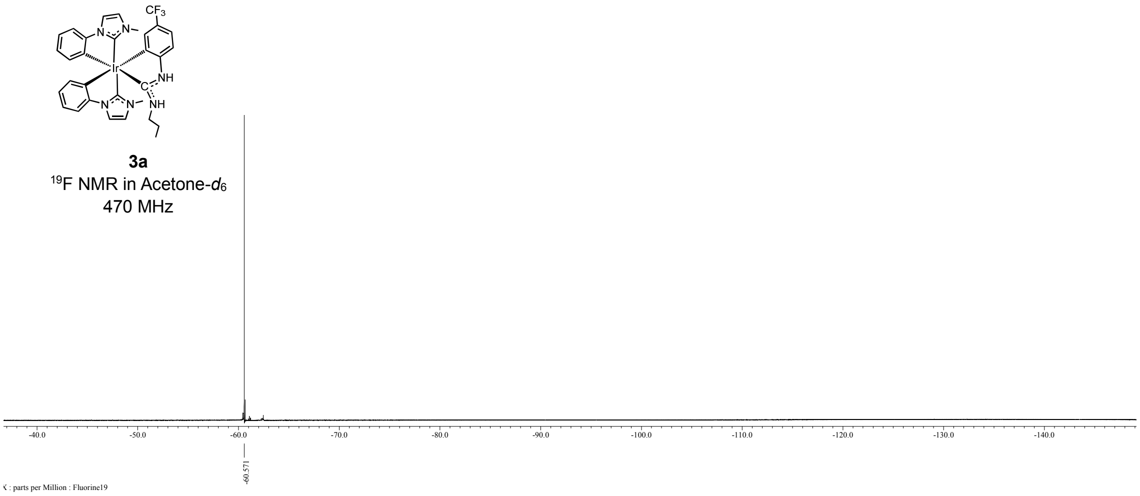

Figure S5. ^{19}F NMR spectrum of **3a**, recorded in acetone-*d6* at 470 MHz.

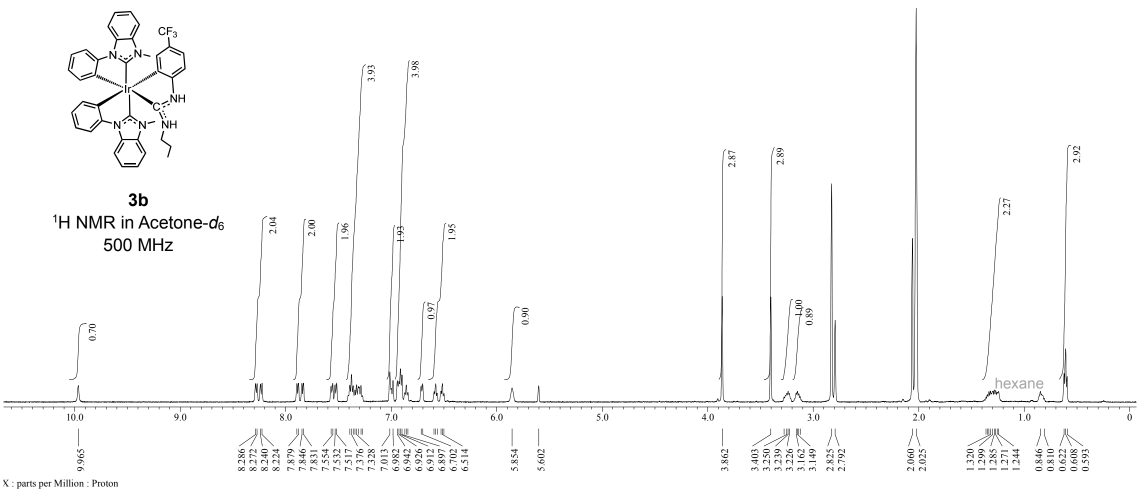

Figure S6. ^{1}H NMR spectrum of **3b**, recorded in acetone-*d6* at 500 MHz.

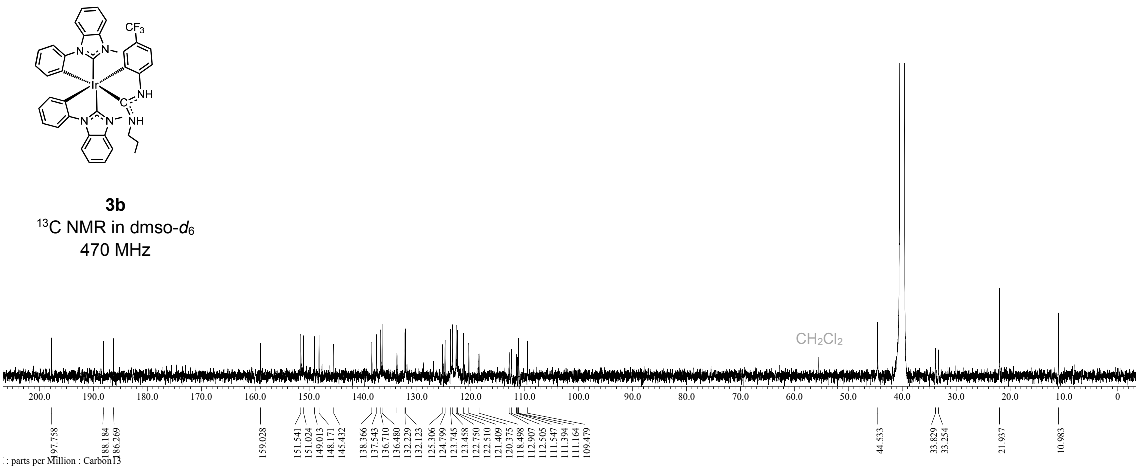

Figure S7. ^{13}C NMR spectrum of **3b**, recorded in dmso-d_6 at 151 MHz.

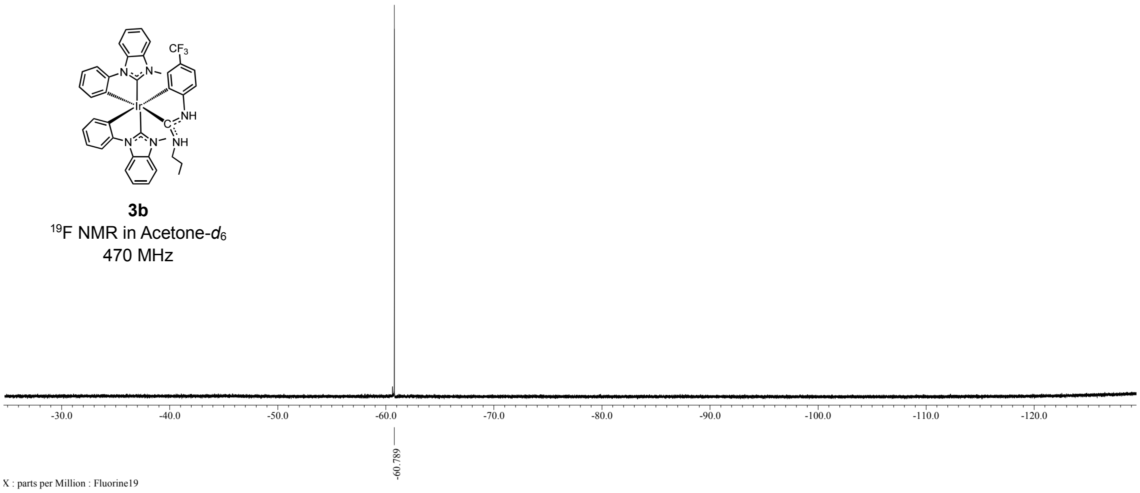

Figure S8. ^{19}F NMR spectrum of **3b**, recorded in acetone-d_6 at 470 MHz.

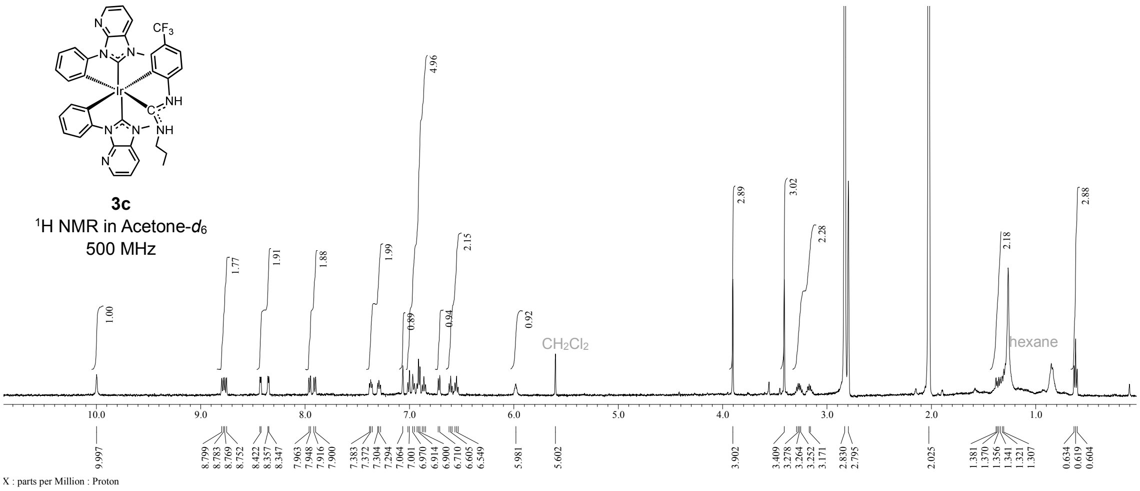

Figure S9. ^{1}H NMR spectrum of **3c**, recorded in acetone-d_6 at 500 MHz.

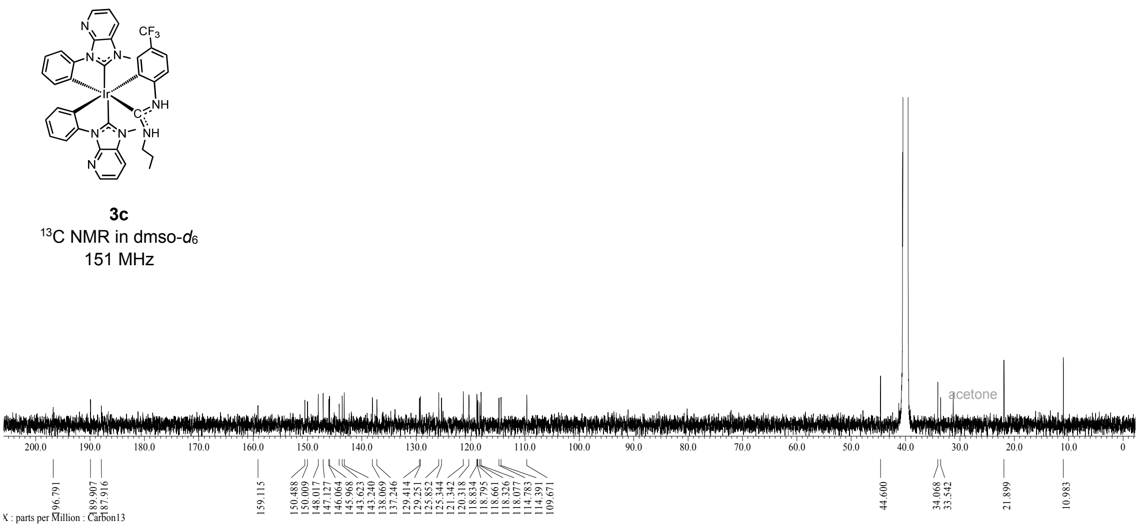

Figure S10. ^{13}C NMR spectrum of **3b**, recorded in dmso-d_6 at 151 MHz. This compound is poorly soluble, including in DMSO, which is the reason for the poor signal-to-noise in this spectrum.

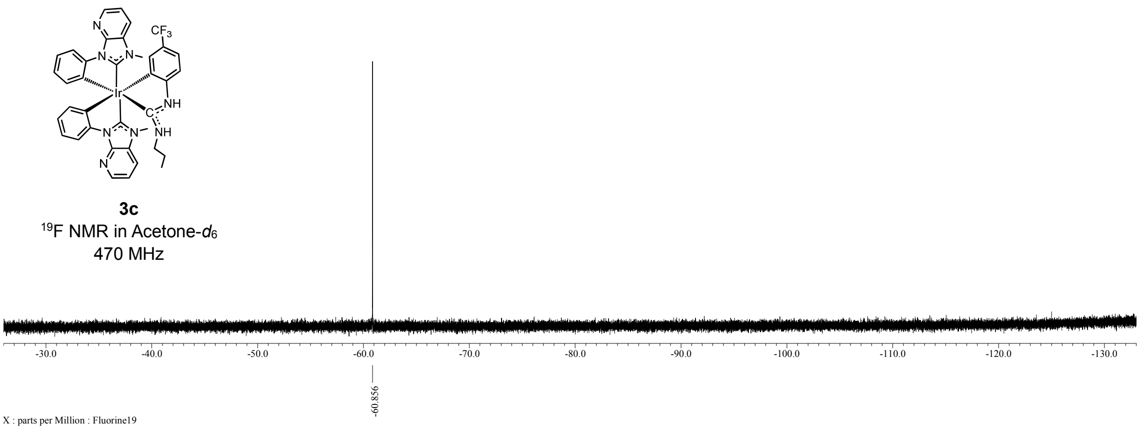

Figure S11. ^{19}F NMR spectrum of **3c**, recorded in acetone-*d*$_6$ at 470 MHz.

Table S2. Optimized Cartesian coordinates for **3a-Me,** in the singlet ground state.

	X	Y	Z
Ir	0.55721	-0.16663	0.07371
C	1.62753	1.57748	0.18619
C	3.68965	2.98062	0.08441
C	0.88866	-0.87238	1.95048
C	1.44795	-1.48649	4.06749
H	1.99981	-1.43281	5.00592
C	0.44318	-2.31388	3.66031
H	-0.05324	-3.13626	4.17255
C	2.72011	0.43427	3.01517
H	3.41396	0.29457	2.16607
H	3.28441	0.38068	3.96056
H	2.24768	1.43036	2.92842
C	-0.73569	-1.81578	0.15346
C	-1.61798	-2.34049	-0.81938
H	-1.61121	-1.92460	-1.84082
C	-2.50543	-3.39734	-0.54098
H	-3.17234	-3.77213	-1.33128
C	-2.54640	-3.97811	0.73471
H	-3.24391	-4.79680	0.95569
C	-1.68147	-3.50589	1.73375
H	-1.69966	-3.95247	2.73885
C	-0.80930	-2.45824	1.41899
C	0.15830	0.05239	-1.91640
C	-0.63049	0.18486	-4.04124
H	-1.33465	0.46591	-4.82458
C	0.55690	-0.48519	-4.09900
H	1.09354	-0.91485	-4.94341
C	-2.07160	1.13830	-2.19209
H	-2.60905	0.43242	-1.53346
H	-2.71115	1.41050	-3.04796
H	-1.82992	2.04140	-1.60634
C	2.15775	-1.20593	-0.80830
C	3.29006	-1.81338	-0.21441
H	3.34291	-1.87669	0.88656
C	4.33612	-2.37122	-0.97602
H	5.19033	-2.84628	-0.47115
C	4.28992	-2.33595	-2.37774
H	5.10248	-2.77090	-2.97478
C	3.19364	-1.73699	-3.02019
H	3.15180	-1.69508	-4.11891
C	2.17228	-1.19586	-2.23079

C	-0.89145	1.13775	0.85874
C	-2.23167	0.93843	1.23606
H	-2.68136	-0.06439	1.13459
C	-3.04176	1.99643	1.70089
C	-2.53289	3.29603	1.83039
H	-3.17237	4.11089	2.18928
C	-1.18942	3.53350	1.50036
H	-0.76356	4.54417	1.60374
C	-0.40999	2.46542	1.03562
C	-4.47868	1.68551	2.02682
N	1.70999	-0.61311	3.01045
N	0.11459	-1.92402	2.36803
N	-0.85322	0.51627	-2.70527
N	1.02720	-0.55251	-2.79334
N	0.95294	2.65386	0.67835
H	1.35316	3.59193	0.73717
N	2.91425	1.77934	-0.17580
H	3.39947	0.93737	-0.48206
F	-5.14479	1.21846	0.94736
F	-4.58971	0.72965	2.97016
F	-5.16110	2.75720	2.47061
H	3.29378	3.85459	-0.47107
H	3.69663	3.23137	1.16822
H	4.72952	2.82140	-0.23947

Point group = C_1

Number of imaginary frequencies = 0

Total electronic energy with ZPE = –1851.27791 a.u.

Table S3. Optimized Cartesian coordinates for the triplet state **3a-Me.**

	X	Y	Z
Ir	0.57840	-0.20497	0.12053
C	1.65381	1.57046	0.20186
C	3.71260	2.96759	0.07422
C	0.92365	-0.92692	1.99343
C	1.45078	-1.53378	4.11874
H	1.98241	-1.47156	5.06816
C	0.47862	-2.38738	3.68665
H	-0.00627	-3.22485	4.18535
C	2.67686	0.44047	3.10384
H	3.40850	0.32137	2.28381
H	3.20306	0.42209	4.07219
H	2.16304	1.41252	2.98431
C	-0.71206	-1.83809	0.18571
C	-1.60872	-2.32944	-0.79216
H	-1.62728	-1.86291	-1.79037
C	-2.47335	-3.40947	-0.53887
H	-3.15200	-3.76363	-1.32815
C	-2.47915	-4.03930	0.71539
H	-3.16092	-4.87622	0.91649
C	-1.60591	-3.59346	1.72033
H	-1.60138	-4.08070	2.70622
C	-0.75529	-2.52178	1.43311
C	0.14069	0.11554	-1.85896
C	-0.66196	0.25983	-3.98104
H	-1.36483	0.56258	-4.75848
C	0.52387	-0.42279	-4.07549
H	1.03180	-0.85592	-4.93437
C	-2.09077	1.20731	-2.13966
H	-2.63185	0.50832	-1.47416
H	-2.73338	1.48103	-2.99287
H	-1.84730	2.11423	-1.55546
C	2.16706	-1.17040	-0.76547
C	3.31700	-1.74641	-0.20298
H	3.40128	-1.79024	0.89635
C	4.32346	-2.36417	-0.99469
H	5.17787	-2.86745	-0.52548
C	4.20485	-2.35921	-2.43537
H	5.00597	-2.81455	-3.03580
C	3.12212	-1.79827	-3.07240
H	3.05491	-1.78351	-4.16853
C	2.09622	-1.19037	-2.25303

C	-0.86173	1.13541	0.89260
C	-2.20011	0.93542	1.27129
H	-2.63324	-0.07827	1.22709
C	-3.03335	2.00981	1.64968
C	-2.54619	3.32352	1.69368
H	-3.20434	4.15087	1.98367
C	-1.20201	3.56024	1.36708
H	-0.79400	4.58261	1.40216
C	-0.40109	2.47620	0.98246
C	-4.47466	1.70023	1.95614
N	1.71169	-0.64912	3.07094
N	0.16937	-2.00123	2.38833
N	-0.88059	0.58199	-2.65337
N	1.01012	-0.54325	-2.76412
N	0.96136	2.66044	0.62385
H	1.35529	3.60284	0.64997
N	2.94493	1.75169	-0.13363
H	3.43767	0.88945	-0.37529
F	-5.11279	1.19145	0.87836
F	-4.59987	0.77629	2.92875
F	-5.17305	2.78170	2.34657
H	3.32809	3.80630	-0.53985
H	3.69473	3.27959	1.14154
H	4.75962	2.79091	-0.21682

Point group = C_1

Number of imaginary frequencies = 0

Total electronic energy with ZPE = –1851.16030 a.u.

References

1 T. Sajoto, P. I. Djurovich, A. Tamayo, M. Yousufuddin, R. Bau, M. E. Thompson, R. J. Holmes and S. R. Forrest, *Inorg. Chem.*, 2005, **44**, 7992–8003.

2 P. Pinter, H. Mangold, I. Stengel, I. Münster and T. Strassner, *Organometallics*, 2016, **35**, 673–680.

3 W. Sattler, L. M. Henling, J. R. Winkler and H. B. Gray, *J. Am. Chem. Soc.*, 2015, **137**, 1198–1205.

4 K. Suzuki, A. Kobayashi, S. Kaneko, K. Takehira, T. Yoshihara, H. Ishida, Y. Shiina, S. Oishi and S. Tobita, *Phys. Chem. Chem. Phys.*, 2009, **11**, 9850–9860.

5 G. M. Sheldrick, *Acta Crystallogr. A*, 2008, **64**, 112–122.

6 A. L. Spek, *Acta Crystallogr. D Biol. Crystallogr.*, 2009, **65**, 148–155.

7 T. Lu and F. Chen, *J. Comput. Chem.*, 2012, **33**, 580–592.
