# Supplementary material for: Mixed-carbene cyclometalated iridium complexes with saturated blue luminescence
Source: Chem Sci. 2019 May 27;10(25):6254–60. doi: 10.1039/c9sc01386e (PMC6617658; doi:10.1039/c9sc01386e)
Supplement: Supplementary file 3 [file SC-010-C9SC01386E-s003.pdf]

GO-LEDs! Mixed carbene cyclometalated iridium complex for smarter OLEDs

The high photoluminescence and color tunability of **cyclometalated iridium** have facilitated its commercial success in organic light-emitting diodes (OLEDs).

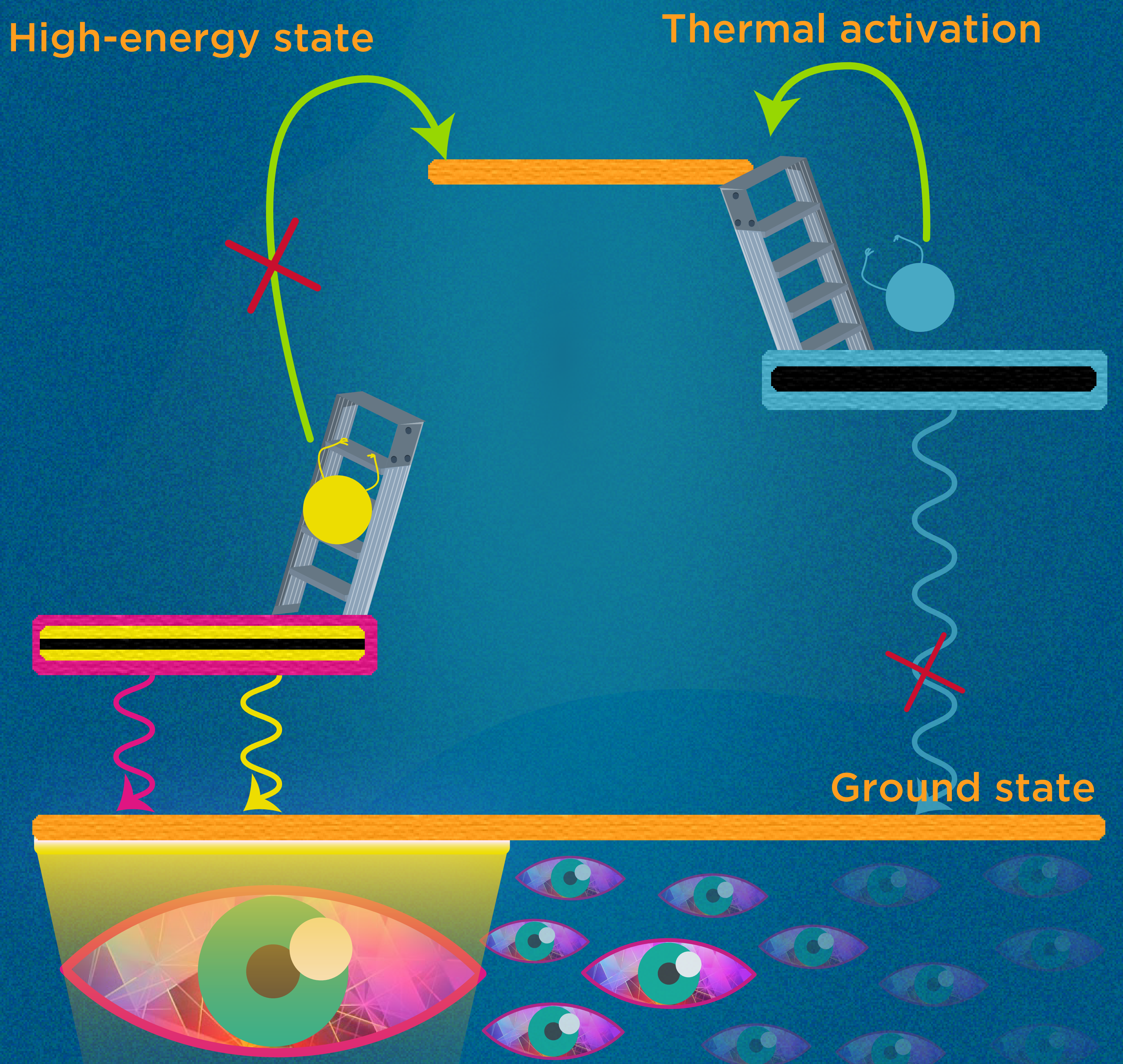

Because the energy of the blue-emitting state is higher, it easily transitions to a non-radiative higher-energy state, thereby resulting in a lack of pure blue emission.

Heteroleptic tris-cyclometalated iridium complexes with mixed carbene

4-trifluoromethylphenyl isocyanide

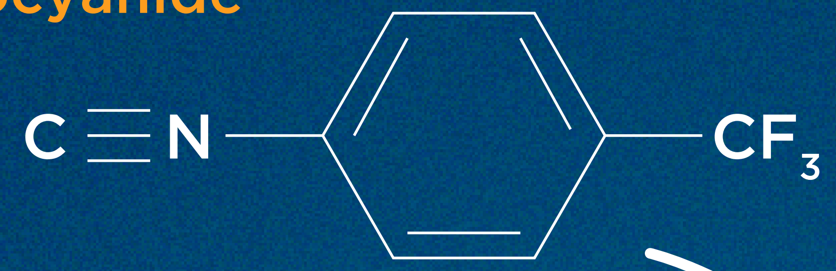

Precursor iridium complex

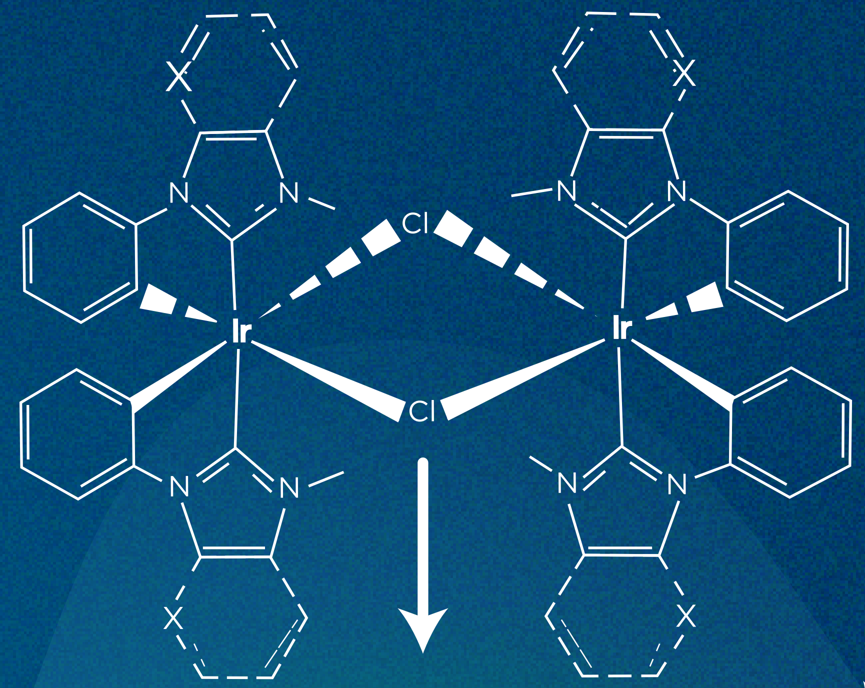

Excess propylamine

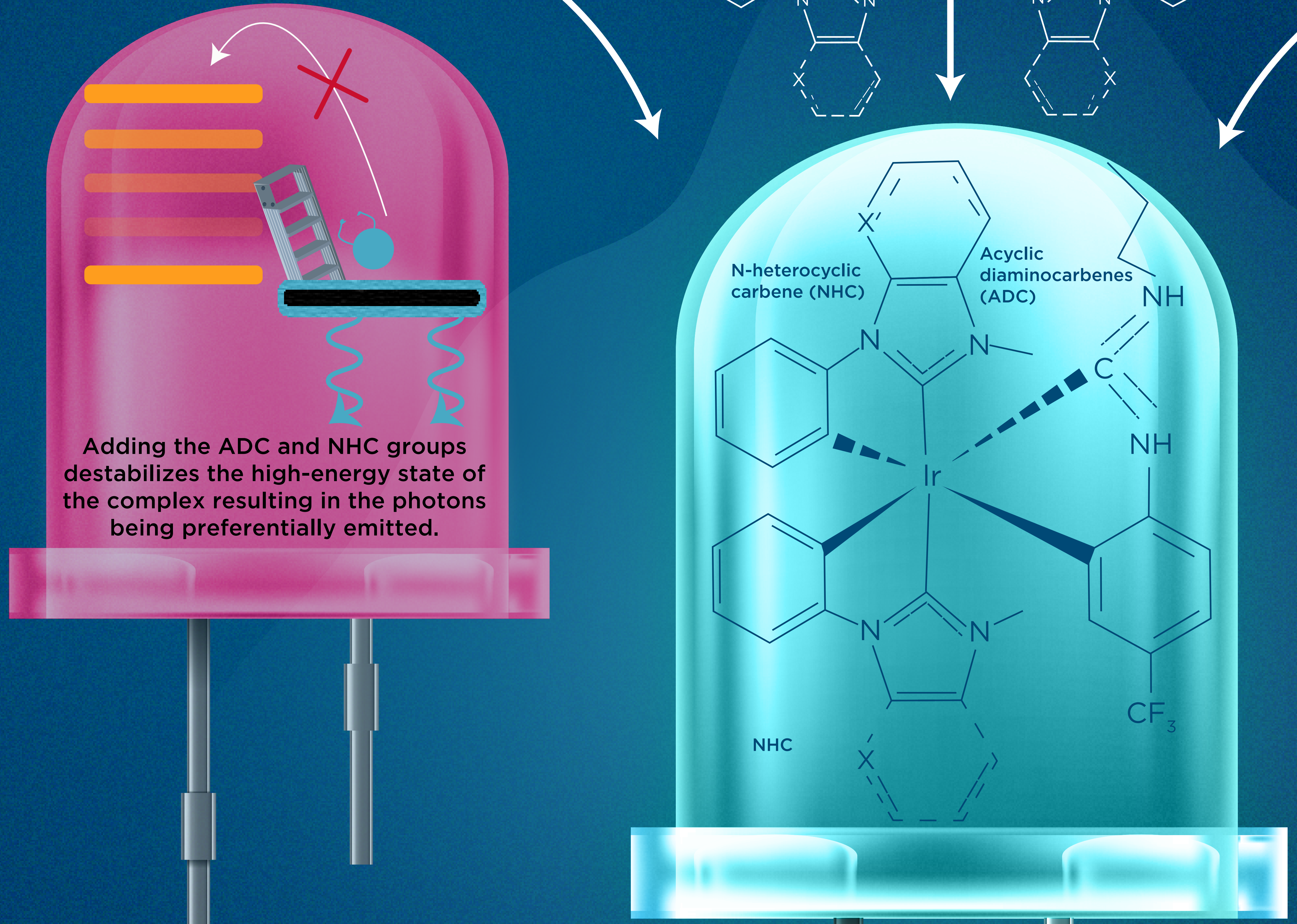

How does it improve OLEDs?

- Rich blue colour
- Matches industry standards
- Efficient photoluminescence
- High stability

The ligand-substituted iridium complex shows promising photoluminescence attributes, which make it an excellent candidate for OLEDs.

Chemical Science

PICK OF THE WEEK

Mixed-Carbene Cyclometalated Iridium Complexes with Saturated Blue Luminescence

Teets *et al.* (2019) | DOI: 10.1039/C9SC01386E

ROYAL SOCIETY OF CHEMISTRY
